# Genetic Diversity of *Lecanosticta acicola* in *Pinus* Ecosystems in Northern Spain

**DOI:** 10.3390/jof9060651

**Published:** 2023-06-09

**Authors:** Nebai Mesanza, Irene Barnes, Ariska van der Nest, Rosa Raposo, Mónica Berbegal, Eugenia Iturritxa

**Affiliations:** 1 Neiker-BRTA, Instituto Vasco de Investigación y Desarrollo Agrario, Granja Modelo s/n, Antigua Carretera Nacional 1, Km. 355, 01192 Arkaute, Spain; 2 Department of Biochemistry, Genetics and Microbiology, Forestry and Agricultural Biotechnology Institute (FABI), University of Pretoria, Pretoria 0083, South Africa; irene.barnes@fabi.up.ac.za (I.B.); ariska.vandernest@fabi.up.ac.za (A.v.d.N.); 3 ICIFOR, INIA-CSIC, Carretera La Coruña Km 7.5, 28040 Madrid, Spain; raposo@inia.csic.es; 4 Instituto Agroforestal Mediterráneo, Universitat Politècnica de València, Camino de Vera S/N, 46022 Valencia, Spain; mobermar@etsia.upv.es

**Keywords:** brown spot needle blight, SSRs, population genetics

## Abstract

*Lecanosticta acicola* is one of the most damaging species affecting *Pinus radiata* plantations in Spain. Favourable climatic conditions and unknown endogenous factors of the pathogen and host led to a situation of high incidence and severity of the disease in these ecosystems. With the main aim of understanding the factors intrinsic to this pathogenic species, a study of the population structure in new established plantations with respect to older plantations was implemented. The genetic diversity, population structure and the ability of the pathogen to spread was determined in Northern Spain (Basque Country), where two thirds of the total *Pinus radiata* plantations of Spain are located. From a total of 153 *Lecanosticta acicola* isolates analysed, two lineages were present; the southern lineage, which was prevalent, and the northern lineage, which was scarce. A total of 22 multilocus genotypes were detected with a balanced composition of both mating types and evidence for sexual reproduction. In addition to the changing environmental conditions enhancing disease expression, the complexity and diversity of the pathogen will make it difficult to control and to maintain the wood productive system fundamentally based on this forest species.

## 1. Introduction

Needle blights are currently the most serious fungal needle diseases affecting pine species worldwide. Among the main causal agents, *Lecanosticta acicola* (Thümen) H. Sydow, *Dothistroma pini* Hulbary and *Dothistroma septosporum* (G. Doroguine) M. Morelet are of particular concern due to the impact they have on *Pinus* ecosystems in the Basque Country, Spain [1]. The symptoms caused by these fungi are quite similar and, therefore, difficult to differentiate, especially when present on the same tree [1]. Severe defoliation is caused by these pathogens that results in significant growth loss when more than 25% of the needles are damaged [2,3]. *Lecanosticta acicola* is considered a regulated non-quarantine pest in the EU since 2019 according to the Commission implementing regulation (EU) 2021/2285 [4].

The disease caused by *Lecanosticta acicola,* brown spot needle blight (BSNB), was well known in Spanish *Pinus radiata* D. Don plantations for decades [5,6]. Until recently, BSNB had only minor impacts on native and exotic forest trees in the north of Spain. This disease was found mainly in valley bottoms, in plantations with high tree density and areas with high humidity. In the past seven years, abnormal climatic conditions favourable to the disease and potentially unknown endogenous factors made the usual silvicultural measures inefficient in mitigating its impact and progress. This pathogen species spread widely, causing severe defoliation and mortality in native *Pinus* forests and plantations in locations where it was not detected before [7,8].

The most characteristic natural pine forests in Spain are those of mountain pine (*Pinus uncinata* Mill. ex Mirb.), Scots pine (*Pinus sylvestris* L.), maritime pine (*Pinus pinaster* Ait.), black pine (*Pinus nigra* Arnold), Aleppo pine (*Pinus halepensis* Mill.) and stone pine (*Pinus pinea* L.); all of them susceptible to *L. acicola* colonisation and damage, with different levels of incidence and severity. *Lecanosticta acicola* was detected in planted *P. sylvestris* and *P. nigra* and other non-native *Pinus* species such as *P. ponderosa* Dougl. ex P. and C. Laws., *P. elliottii* Engelm. and *P. brutia* Ten. [1,9].

*Pinus radiata* is a fast-growing coniferous tree native to the Californian coast and Baja California, in the United States and Mexico, respectively. It is one of the most widely planted tree species in the world, being established in New Zealand, Australia, Chile, South Africa and South-West Europe. In Europe, the most extended covert can be found in the Basque Country in Northern Spain, where the consequences of *L. acicola* impact are very serious for the forestry sector, since *P. radiata* was the basis for the recovery of the wooded area in the region. Its production generates 81% of the total forest products covering 32% of the wooded forest area, as well as constituting 68% of the activity of the wood industry. In this region, this sector is made up of 40,000 people, generates more than 20,000 jobs and accounts for 1.5% of the Basque Country’s GDP, according to data from the Basque Country Competitiveness Report of 2018 [10]. The progress of the disease was exponential since 2010, especially during 2017 and 2019, causing serious defoliation in 33.15% (40,914 Ha) of *Pinus* forest and plantations in the Basque Country. The advance of this disease mainly affects *P. radiata*, although it was also detected in other species. The disease’s impact resulted in the nursery sector ceasing the production of *P. radiata* plants in 2019 and the massive and premature logging of pine severely affected by BSNB, causing serious economic losses for forest owners.

The genetic diversity and structure of the pathogen was studied on a global scale mainly to determine the relationship between North American and European populations [11]. Despite the importance of recent disease outbreaks in Northern Spain, the genetic diversity and population structure of *L. acicola* in the Basque Country is still unknown. Both mating types of *L. acicola* were detected, in some cases even in the same plantation [1], and the presence of the sexual state of this species was confirmed in the Basque Country [12]. Sexual reproduction could result in higher pathogen diversity and adaptation potential. Moreover, ascospore dissemination was associated with long distance dispersal and rapid disease outbreaks in Europe [11]. A high genetic diversity of the pathogen would make the implementation of successful control measures and breeding programs more complicated, and it could enhance the capacity of the pathogen to adapt to changing local conditions [11].

In this context, the importance of knowing the genetic diversity and population structure of the pathogen and its evolutionary potential could help to adapt breeding programs and to design prevention and management strategies against this disease.

## 2. Materials and Methods

### 2.1. Sampling

**Figure 1:**
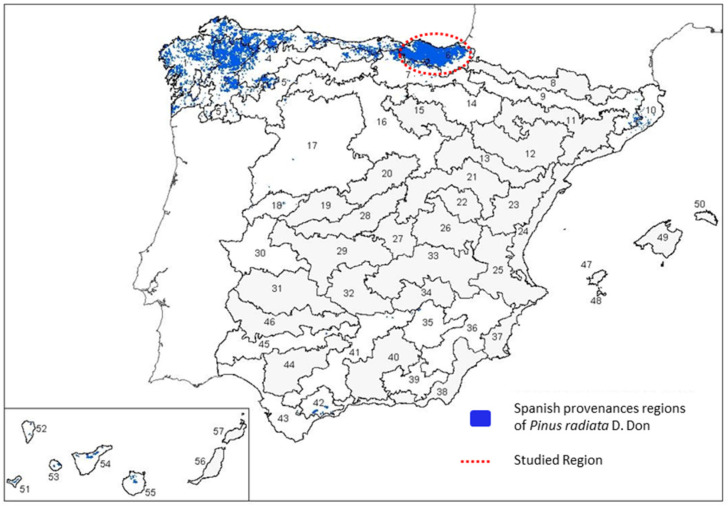
Distribution of provenance regions of radiata pine in Spain. The area marked in red represents the *P. radiata* region of provenance No. 6 and it covers the northern part of the Basque Country (RD 289/2003, Art 2.f. PDF 751 KB; Adapted from https://www.boe.es/eli/es/res/2009/07/28/(3), https://www.miteco.gob.es/es/biodiversidad/temas/recursos-geneticos/geneticos-forestales/rgf_regiones_procedencia.aspx, accessed on 3 April 2023).

This study focused on *Pinus* ecosystems located in the Basque Country, Spanish Atlantic climate region. In the Basque Country, conifers cover an area of 1504.59 km^2^ (20.8%) over 7234 km^2^, of which 1094 km^2^ (15.1% of the total surface) correspond to *P. radiata* (Figure 1). Field observations and sampling were conducted from spring to late autumn in 2018 and 2020.

Three sample collections were differentiated depending on the origin of the plant material. Sample collection 1 (named BC_1) was obtained from 118 different plantations of the Basque Country, representing the infected zones of the *Pinus radiata* provenance No. 6 (Figure 1).

Sample collection 2 (named AR_2) was obtained from 35 needle samples of *Pinus* species (*P. brutia, P. elliottii, P. nigra, P. pinaster, P. pinea, P. ponderosa, P. sylvestris* and *P. taeda*) produced in a French nursery and planted in 2011 in the arboretum AR20 located in Laukiz (Bizkaia) under the European project REINFFORCE (https://reinfforce.iefc.net/es/arboreta/ar20/ accessed on 3 April 2023). AR20 arboretum was established in an abandoned nursery that produced and distributed reproductive material centralising the supply of *P. radiata* to the region under study. Furthermore, this place showed serious needle blight damage. Since this nursery received and grew local (provenance No. 6) and imported seeds (United State, Chile, New Zealand, France, etc.), it was considered to be a potential source of pathogen diversity.

Sample collection 3 (named AR_1) was obtained from 33 symptomatic needle samples from newly established *P. radiata* seedlings in this arboretum. This 2-year-old material (411 seedlings) was established in this location as pathogen trap plants, at a distance of 1.5 m to infected trees from AR_2, to determine the capacity of natural inoculation. 

Needle samples with visible symptoms of BSNB were collected randomly from infected trees at the three sample sites (one sample consist of samples from eight to ten trees from each sampled location) and transported in a cooler box to the laboratory. The majority of samples were collected from *P. radiata* at sample site BC_1, since this was the most prevalent tree species in the studied area. 

### 2.2. Pathogen Isolation

In order to obtain *L. acicola* isolates, needles were examined for typical erumpent fruiting bodies and when possible, five acervuli located on different needles were selected for isolations. Needle surfaces were sterilised by wiping them with a cotton swab soaked with 70% ethanol. Each acervulus was cut from a needle under a dissecting microscope using a scalpel and placed on a glass slide in a drop of sterile water. The presence of typical *L. acicola* conidia was verified under a compound microscope. The conidial suspension was plated on dothistroma selective medium (DSM) with streptomycin using an inoculation loop [13,14]. After four days, germinating conidia were located microscopically on the surface of the medium and transferred to Petri dishes containing DSM to obtain pure, single hyphal cultures [15]. The plates were incubated at room temperature (21 °C). An individual germinated conidium per conidiomata was kept for further analyses. Long term preservation of each fungal isolate was conducted by placing two-week-old mycelium cubes in 10% glycerol at 4 °C and was maintained in the research institute collection (Neiker, Arkaute, Spain).

### 2.3. Pathogen Identification

Fungal tissue was scraped from the surface of 2-week-old cultures with a sterile scalpel blade. The mycelium was homogenised using a Qiagen Tissuelyser II with sterile metal beads (Ø 2.5 mm). DNA was extracted from 100 mg of lysed fungal tissue with the Plant DNA Mini Kit (Analytik Jena AG, Jena, Germany), following the manufacturer’s instructions.

The integrity of the DNA in terms of quality and quantity was verified using a NanoDrop ND-1000 spectrophotometer (Thermo Fisher Scientific, Waltham, MA, USA). DNA working stock solutions of 20 ng/μL were made for polymerase chain reaction (PCR) amplifications. All DNA was stored at −20 °C until further use.

The identity of each isolate was confirmed by species-specific conventional PCR targeting the elongation factor region [16]. The reaction consisted of 0.4 µM of each primer, 10× buffer (Complete II KCl Buffer, IBIAN technologies, Zaragoza, Spain), 200 µM dNTP, 0.5 U IBIAN-*Taq* DNA Polymerase (IBIAN technologies, Zaragoza, Spain), and 1.5 µL DNA template in a total volume of 20 µL. The PCR conditions were as follows: 10 min at 94 °C, 35 cycles of 30 s at 94 °C, 30 s at 60 °C, and 45 s at 72 °C, and a final 10 min extension at 72 °C. PCR amplicons were visualised on a 1% agarose (Conda, Madrid, Spain) gel stained with GelRed^®^ (Biotium Inc., Fremont, CA, USA). The reactions were considered positive for *L. acicola* if an amplicon size of 237 bp was obtained. 

The identification of the isolates was further supported by PCR amplification and sequencing of the internal transcribed spacer (ITS) region, and the translation elongation factor 1-α (*TEF1*) using the primers ITS1 and ITS4 [17], and EF1-728F [18] and EF1-986R, respectively, as described in van der Nest et al. [19]. PCR reactions for each region contained 20 ng DNA, 2.5 μL 10× PCR reaction buffer, 2.5 mM MgCl_2_, 400 nM of each primer, 200 μM of each dNTP and 1 U IBIAN-*Taq* DNA polymerase (IBIAN Technologies, Zaragoza, Spain). The reaction conditions included an initial denaturation step at 94 °C for 10 min, 35 cycles at 94 °C for 30 s, a 45 s annealing step at 56 °C for the ITS region, 52 °C for the *TEF*1 region, 72 °C for 60 s and a final 10 min extension at 72 °C [11] PCR products were purified using the NucleoSpin Gel and PCR Clean-up kit (Macherey-Nagel, Düren, Germany) and sequenced by Eurofins (Genomics, Konstanz, Germany). Sequencing data were edited using Finch TV software version 1.4.0 (https://finchtv.software.informer.com/1.4/, accessed on 17 January 2023) and aligned with MEGA X software version 10.0.4 (https://www.megasoftware.net/, accessed on 17 January 2023). BLAST searches for the fungal taxa were conducted on the NCBI database (National Center for Biotechnology Information NCBI, Bethesda, MD, USA) and the consensus sequences deposited in GenBank. ITS and *TEF1* haplotypes were determined with TCS 1.21 software [20] and nucleotide diversity (Pi) within the Basque Country population was calculated using DnaSP v6 [21].

Phylogenetic trees were inferred using maximum parsimony (MP) and maximum likelihood (ML) analysis in MEGA X. Alignment gaps were set as additional characters with equal value and confidence levels were calculated from 1000 bootstrap replicates. The MP tree was obtained using the Tree-Bisection–Reconnection (TBR) heuristic search option and for the construction of the ML tree, the Hasegawa–Kishino–Yano nucleotide substitution model was used. 

### 2.4. Mating Types Identification

Mating types were identified using specific primers: Md MAT1-1F, Md MAT1-1R, Md MAT1-2F and Md MAT1-2R [22]. PCR conditions consisted of PCR buffer (500 mM KCl, 100 mM Tris-HCL pH 8.8, 0.1% Tween-20, 15 mM MgCl_2_), 200 μM dNTP, 6.4 pmol of each specific primer, 0.5 U *Taq* DNA Polymerase (BIORON GmbH, Ludwigshafen am Rhein, Germany) and 10–20 ng DNA template in a total volume of 20 μL. Reaction conditions included 5 min denaturation at 94 °C, 35 cycles of 30 s at 94 °C, 30 s at 58 °C, 45 s, at 72 °C and a final extension at 72 °C for 7 min [1].

PCR amplicons were visualised on a 2% agarose (Conda, Madrid, Spain) gel stained with GelRed^®^ (Biotium Inc., Fremont, CA, USA). PCR amplicons with a size of 560 bp were scored as MAT 1 and those of 288 bp as MAT 2 [22].

The mating type ratio in each population was calculated. This ratio was expected to be 1:1 for randomly mating populations. A chi-square goodness of fit test for a 1:1 ratio and associated *p* value were estimated to evaluate departure from the null hypothesis (ratio proportion 1:1).

### 2.5. Simple Sequence Repeat (SSR) Loci Amplification and Data Analysis

Ten microsatellite markers MD1, MD2, MD4, MD5, MD6, MD7, MD9, MD10, MD11 and MD12 designed for *L. acicola* were used to amplify the respective regions in the genome [22]. The PCR reaction mixture and reaction conditions with fluorescently labelled primers were carried out as described by Janoušek et al. [11,22]. For fragment analysis, PCR products were pooled into two panels and 1 µL of these multiplexed PCR products was separated on an ABI Prism 3130 Genetic Analyser (Applied Biosystems, Foster City, CA, USA). The mobility of the SSR products was compared to those of the internal size standard, LIZ-500(-250) and allele sizes were estimated by GeneMapper 4.0 computer software (Applied Biosystems, Foster City, CA, USA). A reference sample was run on every gel to ensure reproducibility. 

For each population defined by tree origin, the total number of alleles at each SSR locus was estimated. A multilocus genotype (MLG) was constructed for each isolate by combining data for each of the 10 SSR alleles obtained. The expected multilocus genotype (eMLG) was calculated based on rarefaction using the R package poppr V.2.3.0 [23,24]. Genotypic diversity was conducted for the non-clone-corrected dataset and clone-corrected dataset, in this last case with only one isolate of each MLG considered. Shannon-Wiener index of MLG diversity (H) [25], Stoddart and Taylor’s diversity index (G) [26] and evenness index E5 [27] were calculated using the same R package.

The standardised index of association (rbarD) as an estimate of linkage disequilibrium was calculated to investigate the mode of reproduction [24,28]. The expectation of rbarD for a randomly mating population was zero, and significant deviation from this value would suggest clonal reproduction. Significance was tested based on 1000 permutations and conducted in the R package poppr using the clone-corrected data [24].

The standardised measure of genetic differentiation, G’st, described by Hedrick [29] was calculated to estimate subdivision among populations. This index ranged from 0 to 1, independent of the extent of population genetic variation and locus mutation rates [29]. Pairwise GST values within the clone-corrected data were calculated using the R packages strata G V.1.0.5 [30] and mmod V.1.3.3 [31].

Hedrick’s standardised GST was estimated to assess population structure among these populations [29]. Statistical significance was calculated based on 1000 permutations. Hierarchical analysis of molecular variance (AMOVA) was performed to evaluate the extent of population differentiation and structure among populations, hosts species groups, and within these groups [32].

Discriminant analysis of principal components (DAPC) was performed to infer clusters of populations without considering previous tree origin criteria [33]. DAPC was conducted with the R package adegenet V. 2.0.1 [34] using the Bayesian information criterion (BIC) to infer the optimal number of groups. Important advantages of DAPC are that it maximises variation between the groups, minimises the within-group genetic variability and does not require assumptions regarding evolutionary models [33].

To assess the relationships among MLGs, minimum spanning networks (MSNs) were constructed. Bruvos’s genetic distance matrix and MSNs were generated using the R package poppr V.2.3.0 [23,24]. The genetic distance described by Bruvo et al. [35] takes the SSR repeat number into account, with a distance of 0.1 equivalent to one mutational step (one repeat).

## 3. Results

### 3.1. Isolation and Population Description

A total of 153 isolates were obtained. Taking into account the initial sampling strategy, 97 isolates were obtained from sampling site 1 (BC_1), 28 isolates were obtained from six *Pinus* species from the arboretum AR20 (AR_2) and 28 isolates were obtained from sampling site 3 (AR_1) from seedlings of *P. radiata* planted in late spring of 2020 in this arboretum. These seedlings were obtained from a biosafety P2 greenhouse and the absence of the disease was confirmed by morphological and molecular methods before their establishment in the arboretum. 

### 3.2. Pathogen Identification

**Figure 2:**
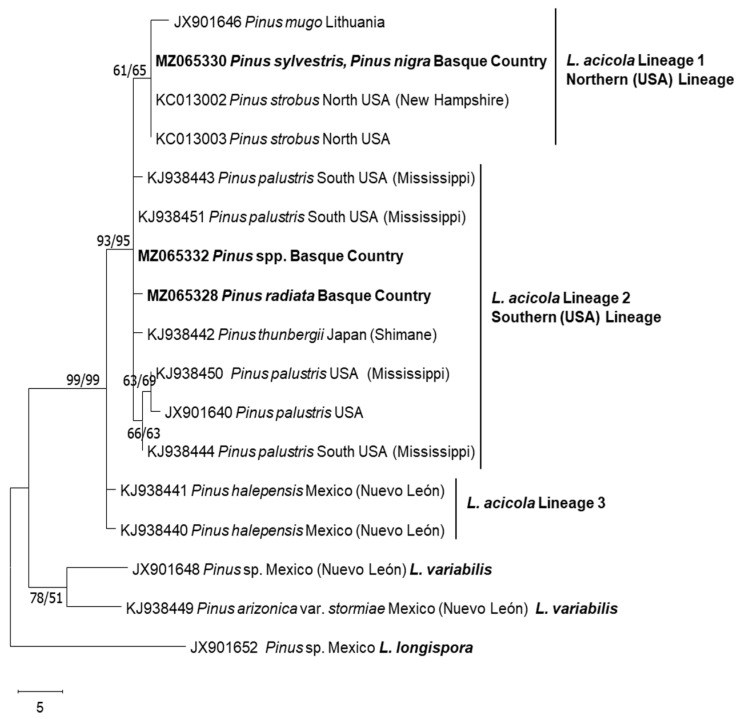
Maximum Parsimony consensus tree of *L. acicola TEF1* sequences. The percentage of replicate trees in which the associated taxa clustered together in the bootstrap test (1000 replicates) are shown next to the branches. The MP tree was obtained using the Tree-Bisection–Regrafting (TBR) algorithm. Maximum parsimony (MP) and maximum likelihood (ML) bootstrap support values (1000 replicates) are indicated at the nodes. Scale bar represents 5 nucleotide mutations. *Lecanosticta longispora* was used as the outgroup.

All 153 isolates were confirmed as *L. acicola* by species-specific conventional PCR targeting the elongation factor region. When analysing the ITS and *TEF1* sequences, only one ITS haplotype was represented by all the isolates, and it was 100% identical (420 aligned nucleotides) to *L. acicola* ex-type KC012999; USA; CMW45427 [36]. Three *TEF1* haplotypes (442 aligned nucleotides) (Figure 2) were distinguished in the Basque Country population with a nucleotide diversity of Pi = 0.00021. Haplotype MZ065328 and haplotype MZ065330 differed from MZ065332 in a single base pair and in two base pairs, respectively. Representative isolates per haplotype were included in the phylogenetic analyses (Figure 2) and deposited into GenBank. These were MZ065328 (representing two isolates: DFA1c06 and DFA5d06), MZ065330 (representing two isolates: h6a25 and h16c25) and MZ065332 (representing 149 isolates). The topologies of the ML and MP phylogenies were similar (Figure 2), where isolates representing the haplotype of MZ065330 were clustered into the northern lineage of *L. acicola* and were identical to the ex-type KC013002 [36]. Isolates of haplotype MZ065328 and MZ065332 were clustered into the southern lineage of *L. acicola*. The haplotype of MZ065332 was 100% identical to KJ938451 (south USA) [11], whereas those of MZ065328 showed a distinctive single base polymorphism.

### 3.3. SSR Loci Data Analysis

All primer pairs amplified the SSR loci in the *L. acicola* Spanish population. Three loci (MD6, MD10, MD11) were monomorphic across all 153 isolates and were, therefore, removed from the analysis (Minor Allele Frequency < 0.01). The Spanish population exhibited a total of 22 MLGs. A clone-correction of the dataset was implemented to remove the bias of resampled MLG in the analysis, resulting in a total of 33 representative isolates (Table 1).

**Figure 3:**
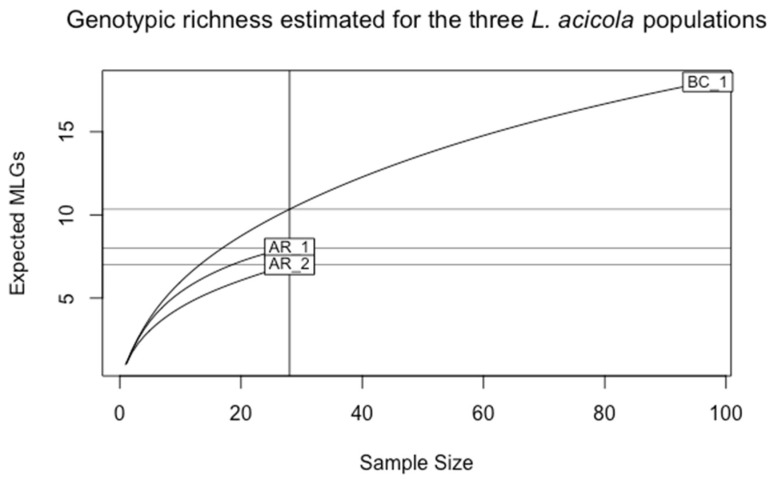
Rarefaction curves showing the genotypic richness of the populations from the Basque Country, BC_1, and the populations from the arboretum, AR_1 and AR_2.

The number of MLGs identified for each sampling site was 18 MLGs for BC_1 and 8 and 7 MLGs, for AR_1 and AR_2, respectively. This difference is related to the sampling size and the number of isolates obtained (N = 97, for BC_1, and N = 28 for AR_1 and AR_2) (Table 1). A more appropriate estimate for richness comparison is the eMLG value, which is an approximation of the number of genotypes that would be expected after correction of the unbalanced sample size based on rarefaction. Thus, genotypic richness was lower in AR_1 and AR_2 compared with BC_1 after sample size correction (Figure 3).

The BC_1 population showed the highest genotypic diversity (G = 18), followed by AR_1 (G = 8) and AR_2 (G = 7) (Table 1). Shannon–Wiener diversity index (H) for the BC_1 population was higher (2.89) than the index for arboretum populations (AR_1 and AR_2). The values of evenness (E5) were the same for the three established populations.

The BC_1 and AR_1 populations showed significant deviation in the rbarD value from the null hypothesis of recombination, not supporting sexual reproduction (rbarD = 0.1798 and rbarD = 0.5678, respectively, with *p* = 0.001 in both cases). On the other hand, AR_2 showed evidence for sexual recombination (rbarD = −0.0656, *p* = 0.796).

**Figure 4:**
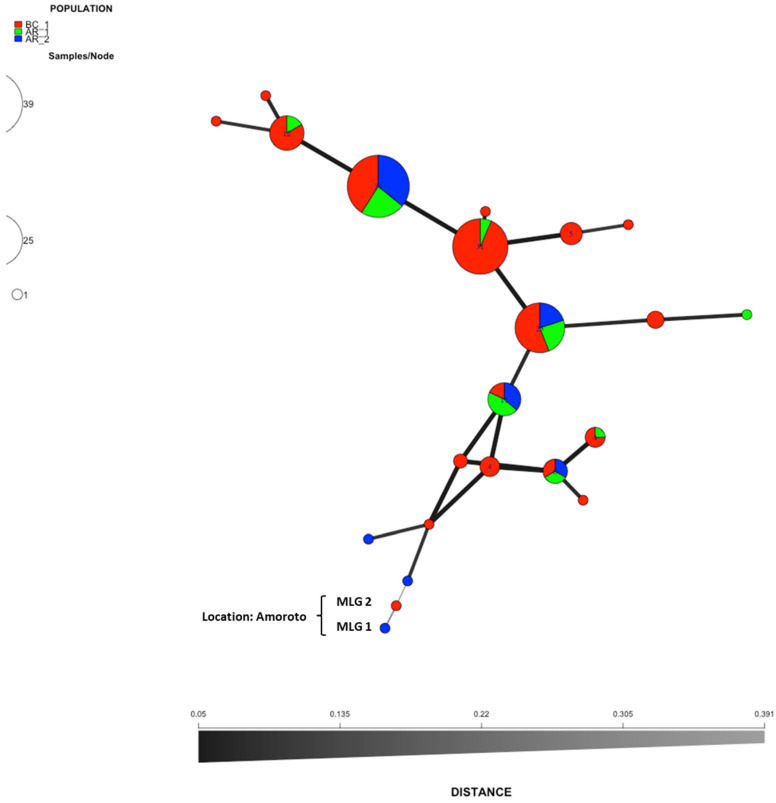
Minimum spanning network from the non-clone-corrected data showing the relationships among the individual multilocus genotypes (MLGs) found among the populations defined by material of origin (BC_1, AR_1 and AR_2). Each node represents a different MLG. Distances and thickness of the lines between nodes are proportional to Bruvo’s distance [35]. Node colours and sizes correspond to the population studied and number of individuals, respectively. MLG1 and MGL2 are the genotypes that show greater distance with respect to the rest, both were located in a 28-year-old *Pinus radiata* plantation in Amoroto (Bizkaia), Spain.

**Figure 5:**
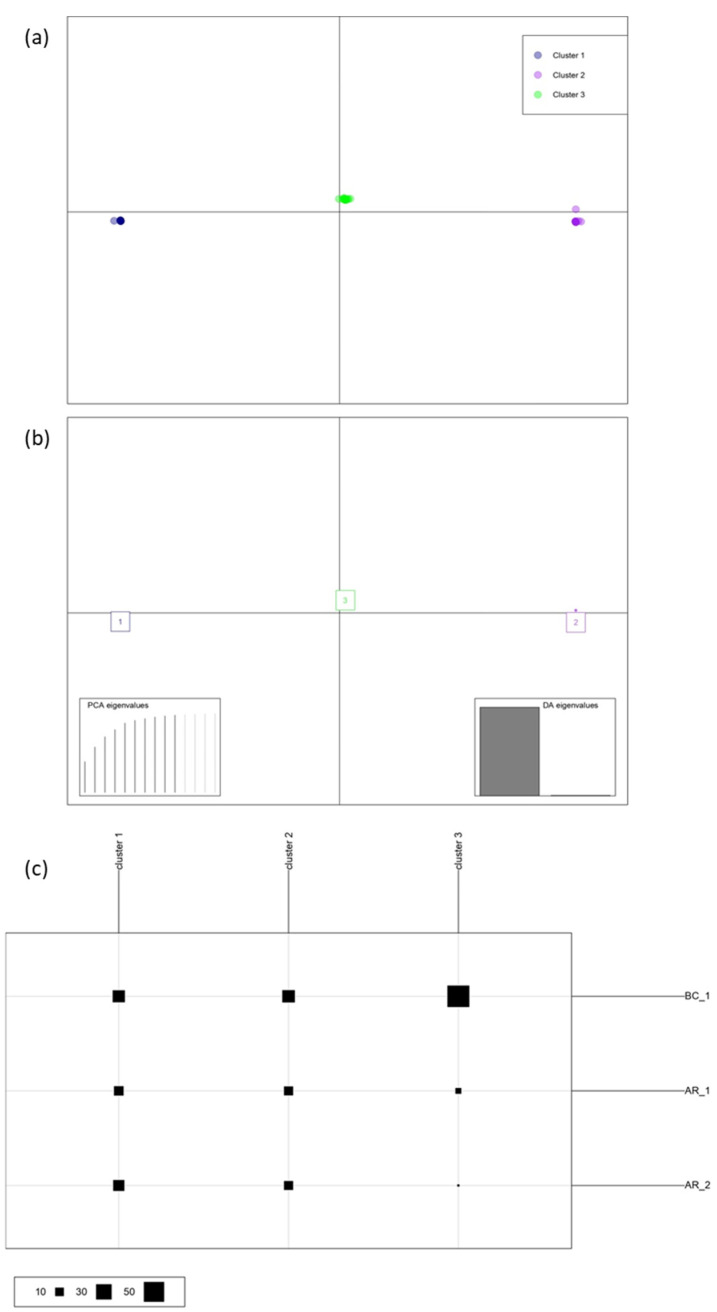
Population structure of the *Lecanosticta acicola* isolates from the Basque Country: (**a**) Scatterplot of the discriminant analysis of principal components for populations BC_1 (blue), AR_1 (purple) and AR_2 (green). The first two discriminant analysis (DA) eigenvalues (highlighted in bottom right hand corner) correspond to the horizontal and vertical axes, respectively. (**b**,**c**) Composition of the DAPC clusters in Figure 4c. Rows correspond to sampled populations while columns correspond to inferred clusters; size of squares in the legend above is proportional to the number of individuals comprising the clusters.

An analysis of molecular variance on the clone–corrected dataset revealed no statistically significant variation among populations (*p* > 0.05, variation within samples *p* = 0.20; variation between samples *p* = 0.24; variation between locations *p* = 0.672). There was no structure in the populations. In BC_1, 7 out of the 22 MLGs identified were present in the population defined by AR location, AR_1 and AR_2, these last populations also showed, respectively, exclusive haplotypes, three in the case of AR_1 and one in the case of AR_2 (Figure 4). The discriminant analysis of principal components (DAPC) chart also showed the lack of population structure between isolates based on location (Figure 5).

Estimated Global Hedrick’s standardised GST per locus (G_ST_ = 0.15), DAPC and AMOVA indicate that there was no population subdivision or population structure defined by locations and sources of samplings.

### 3.4. Mating Identification

Mating type idiomorphs were successfully identified for 151 of the 153 isolates (Table 2). A chi-square test of independence indicated that there was no significant difference (*p* ≤ 0.05) between the mating type ratios observed in the three populations. Both mating types were found in more or less equal proportions except in the AR_1 population, in which Mat 2 was more frequent (Mat-1:Mat-2 = 10:18) (Table 2).

## 4. Discussion

In this study, an intensive sampling of pines in the Basque Country was implemented and *L. acicola* was exclusively detected out of the nine species described in this genus [8]. Only *L. acicola* was reported in Europe within the genus *Lecanosticta* [39] and it was the only species known to cause BSNB until 2022, when *L. pharomachri* was detected in plantations in Colombia causing a severe outbreak of the disease [40].

*Lecanosticta acicola* is currently by far the most damaging and abundant fungal pathogen present in *Pinus radiata* stands in the Spanish provenance region No. 6 together with *Diplodia sapinea* (Fr.) Fuckel 1870 [41]. The reports of *L. acicola* expansion in the Northern Hemisphere increased in the last 15 years, not only in a geographical dimension, but also increasing in the number of host species, and the climatic conditions in which this pathogen is detected [8,39,42]. This emerging disease escalated in incidence and severity in the last decade, affecting the sustainability of *Pinus radiata* ecosystems. In the Basque country, the damage caused by this pathogen accelerated a change in the forest model due to the logging of a thousand hectares a year and the mistrust concerning *P. radiata* sustainability under these circumstances [1].

In the studied area, three *TEF1* haplotypes were identified; one clustered into *L. acicola* northern lineage, identical to the ex-type KC013002 [36] and two clustered into *L. acicola* southern lineage. The northern haplotype represented two isolates and were isolated from seven-year-old *P. sylvestris* and *P. nigra* located in Irisasi (Gipuzkoa). The two haplotypes in the southern lineages included 151 isolates, from which 149 isolates were isolated from *P. radiata*, *P. ponderosa*, *P. nigra*, *P. sylvestris* and *P. brutia*. Two isolates showed a unique base-pair mutation at bp site 101, these were obtained from a *P. radiata* stand located in Amurrio (Araba). In Europe, the southern lineage of *L. acicola* is found in Spain and France and the northern lineage in central and northern Europe [8]. Two isolates out of 153 were part of the northern lineage; however, there are no previous records in Spain of southern and northern lineages coexisting in the same geographical area. Previously, this phenomenon had only been in France [11,39].

The predominance of the southern lineage in the studied area might be due to the northern lineage being a relatively recent introduction. The trees from which the isolates were obtained were produced in two French nurseries located in the Alps of Upper Provence (France) during 2011 and 2012, and in a nursery located in Guémené (France) in 2013, and planted in one of the arboreta established under the European project REINFFORCE. Despite the fact that the material was subjected to phytosanitary controls prior to introduction, it is possible that the pathogen was not detected. Predominance of the southern lineage might also be related to differences in life history traits. For example, southern isolates were reported more virulent to *Pinus* spp. than northern ones with the exception of *P. sylvestris* [43]. Spore germination capacity at 32 °C for the southern isolates was successful, whereas it failed for the northern strains [44] and these differences might contribute to a lack of adaptation to higher temperatures.

Genetic diversity in the BC_1 population obtained from different plantations of the Basque Country was higher compared with the diversity in the arboretum AR20 (location of population AR_1 and AR_2). This arboretum was established in a nursery that centralised the supply of *P. radiata* seedlings of the region under study. This nursery was created to produce and distribute reproductive material to the forest sector. Furthermore, this place showed serious needle blight damage. Since this nursery received and grew local and imported seeds in the past, it was considered to be a potential source of pathogen diversity and dispersal through the seedlings to the entire region. Anthropogenic movement of infected plant material and seedlings is considered the main source of long-distance dispersal of *L. acicola* [8]. The 28 isolates from population AR_2 with eight haplotypes from pine species established twelve years ago, and AR_1 with 7 haplotypes obtained from newly established seedlings in the arboretum, support the hypothesis of their high and fast colonisation capacity of different hosts but mainly *P. radiata*, which shows a high susceptibility to disease in the region [1].

Indication of sexual recombination in the sampled region was supported by the fact that both mating types were identified in more or less equal proportions in the populations of *L. acicola*, the high levels of observed genetic diversity, and by many of the isolates with the same multilocus haplotypes having different mating types in the same populations. Direct evidence for the sexual state of the pathogen already exists in a location 0.53 km far away from the arboretum [12] and it may also be present in these areas. Population structure analysis showed no evidence of population subdivision. It is likely that there is only one panmictic population present throughout all locations. However, a more intensive sampling of these areas may reveal new hypotheses about population structure, as was observed in other fungal species in the region [41].

The seeds and plants used in Northern Spain come from distributors in the United States, France, Denmark, New Zealand, Chile, etc., which makes it difficult to generate hypotheses about the potential origin of the pathogen’s introduction [45]. Nevertheless, considering that the main pine species in the studied region is *Pinus radiata* and that the pathogen is absent in Chile and New Zealand, either the United States and/or France could be the main candidates for the pathogen introduction into Spain. Previous population analysis established that the origin of the northern and southern lineages present in Europe were from North America [11,46,47]. The Basque Country was the first location in Europe where the presence of *L. acicola* was confirmed, and where North America was potentially considered the source of the infected host plants [39]. The presence of the isolates from the northern lineage in our area could be a relatively newer introduction from other European countries caused by the northern lineage spreading within Europe through separate introductions, and thus defining characteristic populations [46,47]. 

The knowledge of the origin, diversity and genetic structure of pathogen populations at a global and local scale can have a remarkable impact on landscape-level planning models and other decision support systems that enable forest managers to generate optimal disease management strategies. The high levels of genetic diversity of the pathogens would complicate the implementation of successful control measures and breeding programs, and could enhance the capacity of adaptation of the pathogen to stressful conditions. In this context, preventative methods should be directed to reduce the movement of plants among countries and regions to avoid the introduction of new genetic sources of diversity into existing populations. This is even more important now in Spain, seeing that a possible recent introduction of a northern lineage was discovered in this study and that adaptation of isolates in each lineage to local climatic conditions could contribute to the success of the pathogen [8]. Some areas are so devastated by the disease that restrictions in the use of highly susceptible pine species might need to be restricted in plantations to help reduce inoculum pressure and the species becoming reservoirs of the pathogen.

## Figures and Tables

**Table 1:** Genetic diversity indices and linkage disequilibrium based on the standardised index of association (rbarD) of *Lecanosticta acicola* populations defined by isolates geographic origin (BC_1, AR_1 and AR_2).

Parameters	Non-Clone-Corrected Dataset				Clone-Corrected Dataset			
Pop	BC_1	AR_2	AR_1	Total	BC_1	AR_2	AR_1	Total
N	97	28	28	153	18	7	8	33
MLG	18	7	8	22	18	7	8	22
eMLG	10.3	7	8	10.1	10	7	8	8.81
SE	1.55	0	0	1.6	0		0	0.90
H	2.24	1.48	1.81	2.28	2.89	1.95	2.08	2.97
G	6.4	3.21	5.03	6.71	18	7	8	17.3
E.5	0.642	0.654	0.791	0.649	1	1	1	0.88
rbarD	0.149	0.434	0.107	0.189	0.1798	0.5678	−0.0656	0.2599
*p*-value	0.001	0.001	0.001		0.001	0.001	0.796	

Abbreviation of Statistic: Pop, population name. N, number of individuals observed. MLG, number of multilocus genotypes observed. eMLG, the number of expected MLG at the smallest sample size ≥ 7 based on rarefaction analysis. SE, standard error based on eMLG. H, Shannon–Wiener Index of MLG diversity [25]. G, Stoddart and Taylor’s Index of MLG diversity [26]. E5, evenness [27,37,38], rbarD, the standardised index of association [28] and *p*-value based on rbarD index.

**Table 2:** Mating type ratios in the three populations of *Lecanosticta acicola*.

Population	N	Mating-Type Ratio (Mat-1:Mat-2)	X^2^	*p*
BC_1	97 *	55:40	2.36	0.124
AR_1	28	10:18	2.28	0.131
AR_2	28	14:14	0.00	1
Total	153	79:72	0.33	0.569

* The mating type could not be determined for two of the isolates.
